# Association of Circulating Brain-Derived Neurotrophic Factor With Coronary Risk Factors and Coronary Artery Disease in Patients Who Have Undergone Coronary Computed Tomography Angiography

**DOI:** 10.14740/cr2201

**Published:** 2026-06-05

**Authors:** Tetsuro Tachibana, Yuhei Shiga, Erika Miura-Takahashi, Kohei Tashiro, Riku Tsudome, Yuto Kawahira, Takashi Kuwano, Makoto Sugihara, Satoshi Imaizumi, Shin-ichiro Miura

**Affiliations:** a Department of Cardiology, Fukuoka University School of Medicine, Fukuoka, Japan

**Keywords:** Brain-derived neurotrophic factor, Computed tomography angiography, Coronary artery disease

## Abstract

**Background:**

Previous studies have reported low circulating brain-derived neurotrophic factor (BDNF) concentrations in patients with coronary artery disease (CAD), but findings have been inconsistent and may be influenced by platelet-related factors. This study evaluated the association between plasma BDNF and CAD in patients undergoing coronary computed tomography angiography (CCTA).

**Methods:**

We prospectively enrolled 402 consecutive patients who underwent CCTA for CAD screening at Fukuoka University Hospital and who were either clinically suspected of having CAD or had at least one cardiovascular risk factor. CAD was defined as coronary artery stenosis of 50% or greater. Plasma BDNF was measured and its association with CAD and clinical variables was examined using multivariate linear regression and logistic regression. The effect of covariate adjustment on the association between BDNF and CAD was assessed using the change-in-estimate method.

**Results:**

Patients with CAD (n = 186) had lower plasma BDNF levels compared with patients without CAD (n = 216) (5.73 ± 3.16 vs. 6.41 ± 3.42 ng/mL, P = 0.039). Multivariate linear regression analysis revealed that platelet count was the strongest determinant of plasma BDNF levels (β = 0.027, P < 0.001). Unadjusted logistic regression analysis showed that higher BDNF levels were associated with a lower prevalence of CAD (odds ratio (OR) 0.94, 95% confidence interval (CI) 0.88–1.00, P = 0.039). However, after adjusting for platelet count, this association weakened and became non-significant (OR 0.976, 95% CI 0.909–1.05, P = 0.49). Further adjustment for age and coronary artery calcification score did not significantly alter the results.

**Conclusions:**

In a cohort undergoing CCTA with a relatively intermediate pre-test probability of CAD, plasma BDNF levels were lower in patients with CAD, but this crude correlation was primarily explained by platelet-related confounding factors. Platelet count should be considered when evaluating BDNF as a cardiovascular biomarker in patients with intermediate cardiovascular risks.

## Introduction

Coronary artery disease (CAD) remains a major cause of mortality worldwide. Established clinical risk factors include dyslipidemia (DLP), diabetes mellitus (DM), smoking, hypertension (HT), obesity, and a family history of CAD [1]. Coronary computed tomography angiography (CCTA) is widely used in Japan for noninvasive assessment of coronary stenosis, calcification, and plaque [2, 3], and provides high diagnostic accuracy with a sensitivity of 89% and specificity of 96% [4, 5]. Using the Fukuoka University CCTA registry (FU-CCTA), we have been investigating risk factors related to the primary prevention of CAD [6–9].

Brain-derived neurotrophic factor (BDNF) is a dimeric polypeptide best known for its roles in neuronal differentiation, survival, and synaptic plasticity [10–12]. Beyond its neurological functions, BDNF has been implicated in biological processes relevant to coronary heart disease, including lipid metabolism, atherosclerotic plaque formation, inflammation, and regulation of adhesion molecules [13]. Higher BDNF levels have been associated with increased body mass index (BMI), blood pressure, low-density lipoprotein cholesterol (LDL-C), and total cholesterol, while simultaneously being linked to reduced cardiovascular events and mortality, independent of BMI and physical activity [14, 15].

Meta-analyses have reported lower circulating BDNF levels in patients with CAD [16]. However, the relationship between BDNF and cardiovascular risk factors remains controversial, as both protective and adverse associations have been described [17, 18].

Therefore, we examined BDNF levels in patients undergoing CCTA and evaluated the association between BDNF and the presence of CAD, and the relationship between BDNF and conventional cardiovascular risk factors.

## Materials and Methods

### Study subjects

A prospective cohort study was conducted. We enrolled 402 consecutive patients who underwent CCTA for screening of CAD at Fukuoka University Hospital and either were clinically suspected of having CAD or had at least one cardiovascular risk factor. The use of CCTA was determined in general clinical practice. Patients with a high pre-test probability of CAD may undergo coronary angiography (CAG) or functional stress testing instead of CCTA. Therefore, the present cohort represents a clinically referred population rather than an unselected population. The study was approved by the Fukuoka University Ethics Committee (09-10-02) and was registered at the UMIN Clinical Trials Registry (#000016641). All the participants were provided with the informed consent form before enrollment and all the protocol was performed according to the Declaration of Helsinki (Fortaleza, October 13, 2013).

### Coronary artery evaluation

Coronary artery stenosis was evaluated by constructing a volume-rendered image using Aquilion ONE, a slice CT manufactured by Canon, and evaluating the degree of lumen stenosis with multiplanar images. Using CCTA, ≥ 50% of coronary stenosis was considered to be CAD according to the Society of Cardiovascular Computed Tomography Guidelines [19]. The severity of coronary artery stenosis was determined by the number of lesion branches, the coronary artery calcification (CAC) score, and the Gensini score [20]. The cardiac function analysis function installed in Aquilion ONE was used to automatically trace and analyze both diastolic and systolic cardiac function, and left ventricular mass (LVM), ejection fraction (EF), left ventricular end diastolic volume (LVEDV), and left ventricular end systolic volume (LVESV) were measured. The LVM index (LVMI) was calculated by dividing the LVM by the body surface area (BSA) of each patient. BSA was calculated by Du Bois formula (height (m)^0.725^ × weight (kg)^0.425^ × 0.007184) [21].

### Cardiovascular risk assessment

Patient characteristics included age, sex, BMI, presence or absence of coronary risk factors (family history of CAD, smoking history, DM, HT, DLP), blood pressure, complete blood count, triglyceride (TG), high-density lipoprotein cholesterol (HDL-C), LDL-C, non-HDL-C, hemoglobin A1c (HbA1c), medication history, and presence or absence of CAD and number of affected branches as determined by CCTA. Blood high-sensitivity C-reactive protein (hs-CRP) levels were also measured. Brachial-ankle pulse wave velocity (baPWV, value for both sides) was measured. All patients were evaluated with regard to chronic kidney disease (CKD), and estimated glomerular filtration rate (eGFR) was assessed. Metabolic syndrome (MetS) was defined as at least two of a visceral fat area of 100 cm^2^ or more, TG of 150 mg/dL or more or HDL-C of 40 mg/dL or less, fasting blood glucose of 110 mg/dL or more, systolic blood pressure of 130 mm Hg or more, and a diastolic blood pressure of 85 mm Hg or more [22]. Symptom data were not systematically collected for the present analysis. Coronary microvascular dysfunction and vasospastic angina were not specifically assessed.

### Measurement of BDNF

Plasma levels of BDNF (Cat. #DBD00) were determined in duplicate by specific enzyme immunoassays according to the manufacturer’s instructions (R&D Systems, Minneapolis, MN).

### Medication

Medication use was recorded by category. Medications for HT included an angiotensin-converting-enzyme inhibitor/angiotensin II receptor blocker (ACEi/ARB), beta-blocker, calcium channel blocker (CCB), or diuretic (DU). Medications for DLP included statins or eicosapentaenoic acid (EPA). Medications for DM included α-glucosidase inhibitor (α-GI), biguanide, dipeptidyl peptidase-4 inhibitor (DPP-4i), insulin, sulfonylurea (SU), or thiazolidinedione.

### Statistical analysis

Statistical analyses were performed using Excel (SSRI, Tokyo, Japan) at Fukuoka University (Fukuoka, Japan) and EZR (Jichi Medical University Saitama Medical Center, Saitama, Japan), a graphical user interface for R (The R Foundation for Statistical Computing, Vienna, Austria). Continuous variables are presented as mean ± standard deviation (SD). Categorical and continuous variables were compared between groups using Chi-square tests and *t*-tests, respectively. For BDNF, between-group comparisons were performed using the presence of CAD, the number of lesions, and the presence of multivessel disease as variables. Furthermore, the relationship between BDNF and predictors (risk factors for atherosclerotic cardiovascular disease) was analyzed using linear regression analysis. Independent variables were identified using univariate and multivariate analyses for predictors of CAD presence. A logistic regression model was used to evaluate multiple independent variables with the presence of CAD as the dependent variable. Independent variables were selected from factors thought to have a clinical impact on the presence of CAD (risk factors for atherosclerotic cardiovascular disease) and factors that showed significant differences in univariate analysis, including BDNF, the focus of this study. Model complexity was considered in relation to the number of CAD events. Covariates were selected based on clinical relevance and variables showing group differences and potential confounding effects.

## Results

### Patient characteristics at baseline for all patients in the CAD and non-CAD groups

Patient characteristics for the non-CAD and CAD groups are shown in Table 1. A total of 402 patients were enrolled: 216 in the non-CAD group and 186 in the CAD group. The mean age was 63 ± 14 years in the non-CAD group and 69 ± 10 years in the CAD group, showing a significant difference. Regarding gender, the male ratio was 40.7% in the non-CAD group versus 60.2% in the CAD group, showing a significant difference (P < 0.01). Similarly, significant differences were observed in smoking rate (27.8% vs. 38.2%, P < 0.05), DM (21.3% vs. 33.5%, P < 0.05), and DLP (63.4% vs. 75%, P < 0.05). Blood tests showed significant differences in HDL-C (61.4 ± 15.8 vs. 56.7 ± 16.4 mg/dL, P < 0.01), eGFR (70.3 ± 14.2 vs. 65.9 ± 14.9 mL/min/1.73 m^2^, P < 0.01), HbA1c (5.9±0.72% vs. 6.1±0.84%, P < 0.01), and platelet (237.2 ± 57.8 vs. 202.6 ± 52.1 × 10^3^/µL, P < 0.05). No significant differences were observed for hs-CRP or LDL-C. Regarding cardiac function, significant differences were observed for EF and LVMI.

**Table 1:** Patient Characteristics at Baseline for All Patients in the CAD and Non-CAD Groups

	Non-CAD group (n = 216)	CAD group (n = 186)	Non-CAD vs. CAD group, P-value
Age (years)	63 ± 14	69 ± 10	< 0.001
Gender (male) (%)	40.7	60.2	< 0.001
Body mass index (kg/m^2^)	23.6 ± 3.9	23.9 ± 3.9	0.419
Family history of CAD (%)	20.8	18.3	0.532
Smoking (%)	27.8	38.2	0.033
Hypertension (%)	60.7	58.6	0.685
Diabetes mellitus (%)	21.3	33.5	0.005
HbA1c (%)	5.9 ± 0.7	6.1 ± 0.8	0.005
FBG (mg/dL)	105 ± 33	113 ± 31	0.025
Dyslipidemia (%)	63.4	75	0.023
TG (mg/dL)	133 ± 123	136 ± 104	0.832
HDL-C (mg/dL)	61 ± 16	57 ± 16	0.005
LDL-C (mg/dL)	118 ± 33	114 ± 34	0.347
TC (mg/dL)	208 ± 38	202 ± 40	0.125
Hb (g/dL)	13.8 ± 1.5	14.0 ± 1.6	0.273
Plt (10^3^/µL)	237.2 ± 57.8	202.6 ± 52.1	0.03
hs-CRP	0.14 ± 0.43	0.14 ± 0.32	0.869
BDNF (ng/mL)	6.41 ± 3.42	5.73 ± 3.16	0.039
Chronic kidney disease (%)	26.8	26.9	1
eGFR (mL/min/1.73 m^2^)	70 ± 14.2	66 ± 14.8	0.002
Metabolic syndrome (%)	24.1	34.9	0.052
EF (%)	65.9 ± 7.3	63.2 ± 9.6	0.002
LVMI (g/m^2^)	61.2 ± 18.1	68.2 ± 22.2	< 0.001
ASO (%)	3.2	7	0.108
Lt. PWV	1510 ± 318	1698 ± 379	< 0.001
CAC score	38 ±162	458 ±773	< 0.001
Medications			
ACEi/ARB (%)	28.7	38.7	0.044
CCB (%)	31.5	48.9	< 0.001
β-blocker (%)	5.6	10.8	0.065
DU (%)	5.1	8.1	0.309
Statin (%)	24.1	37.1	0.006
EPA (%)	4.2	1.6	0.154
SU (%)	2.8	8.6	0.014
α-GI (%)	1.9	2.2	1
Biguanide (%)	5.6	5.9	1

Continuous variables are expressed as mean ± SD. Significance level was set at P < 0.05. α-GI: α-glucosidase inhibitor; ACEi/ARB: angiotensin-converting-enzyme inhibitor/angiotensin II receptor blocker; ASO: arteriosclerosis obliterans; BDNF: brain-derived neurotrophic factor; CAC: coronary artery calcification; CAD: coronary artery disease; CCB: calcium channel blocker; DU: diuretic; EF: ejection fraction; eGFR: estimated glomerular filtration rate; EPA: eicosapentaenoic acid; FBG: fasting blood glucose; Hb: hemoglobin; HbA1c: hemoglobin A1c; HDL-C: high-density lipoprotein cholesterol; hs-CRP: high-sensitivity C-reactive protein; LDL-C: low-density lipoprotein cholesterol; LVMI: left ventricular mass index; Plt: platelet; PWV: pulse wave velocity; SU: sulfonylurea; TC: total cholesterol; TG: triglyceride.

Coronary artery evaluation showed significant differences in the number of diseased vessels, CAC score, and Gensini score. Regarding treatment, significant differences were observed in ACEi/ARB, CCB, statin, SU, and α-GI use.

### BDNF profile at baseline for all patients in the CAD and non-CAD groups

**Figure 1:**
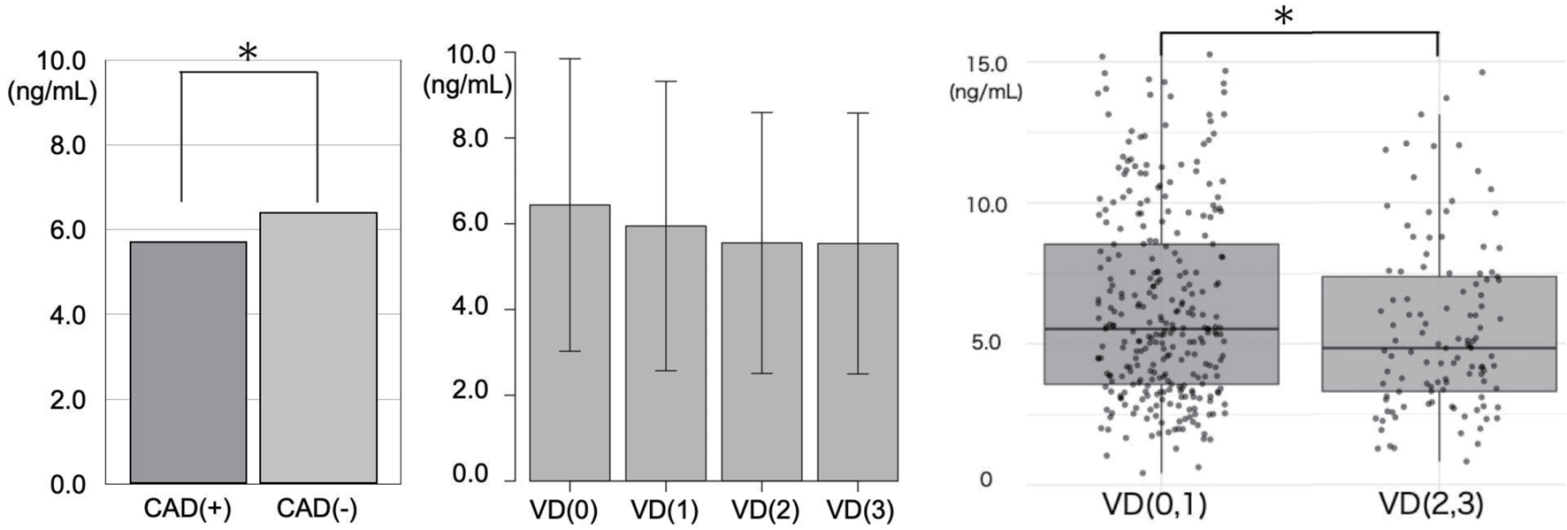
BDNF profile at baseline for all patients in the CAD and non-CAD groups or in each the number of VD. *P < 0.05. BDNF: brain-derived neurotrophic factor; CAD: coronary artery disease; VD: the number of significant vessel diseases.

BDNF profile at baseline for all patients is shown in Figure 1. In all patients, BDNF was 6.10 ± 3.33 ng/mL. BDNF in the non-CAD group was 6.41 ± 3.42 ng/mL, while in the CAD group it was 5.73 ± 3.16 ng/mL, and the difference was significant. The relationship between the number of significant vessel diseases (VD) and BDNF was analyzed using one-way analysis of variance (ANOVA), but no significant differences were found between groups (Fig. 1). When categorized by the presence of multi-vessel disease (VD = 0–1 or VD = 2–3), BDNF was significantly higher in the VD = 0–1 group (6.3 ± 3.4 ng/mL, n = 289) than in the VD = 2–3 group (5.6 ± 3.0 ng/mL, n = 113) (P = 0.03) (Fig. 1).

### Multivariable linear regression analysis of BDNF

The results of the multivariable linear regression analysis are shown in Table 2. A multivariate linear regression model was constructed with BDNF (ng/mL) as the dependent variable, incorporating both continuous and categorical cardiovascular risk factors (Table 2). The categorical variables incorporated into the model were CAD, CKD, DLP, DM, family history of CAD, HT, sex, smoking status, and number of diseased vessels (VD). Continuous variables were age, BMI, CAC score, hs-CRP, LVMI, and PWV.

**Table 2:** Linear Regression Analysis of BDNF With CAD and Cardiovascular Risk Factors

Variables	Estimate	95% CI	P-value
Plt	0.027	0.021–0.032	< 0.001
Hypertension	0.567	−0.043 to 1.178	0.069
Family history	0.58	−0.175 to 1.335	0.132
Smoking	0.507	−0.185 to 1.199	0.15
Chronic kidney disease	0.489	−0.193 to 1.170	0.159
Age	−0.01	−0.042 to 0.021	0.518
Male	−0.217	−0.982 to 0.549	0.578
Dyslipidemia	−0.145	−0.805 to 0.515	0.667
Lt. PWV	0	−0.001 to 0.001	0.772
Diabetes mellitus	−0.041	−0.732 to 0.650	0.908
CAC score	0	−0.001 to 0.001	0.926
CAD	0.051	−1.121 to 1.223	0.932
hs-CRP	0.277	−0.494 to 1.047	0.481

Significance level was set at P < 0.05. BDNF: brain-derived neurotrophic factor; CAC: coronary artery calcification; CAD: coronary artery disease; CI: confidence interval; hs-CRP: high-sensitivity C-reactive protein; Lt. PWV: left pulse wave velocity; Plt: platelet.

Among all covariates, platelet count was independently associated with BDNF (β = 0.0266, 95% confidence interval (CI) 0.021–0.032, P < 0.001). Increased platelet count was strongly associated with elevated BDNF. Cardiovascular risk factors including DM, CAD, CKD, HT, DLP, sex, and smoking status did not show statistically significant associations with BDNF. Age, BMI, CAC score, hs-CRP, LVMI, baPWV, and VD did not show significant associations with BDNF.

### Multivariate logistic regression analysis of risk factors for CAD

The results of the multivariable logistic regression analysis are shown in Table 3.

**Table 3:** Multivariable Analyses of Predictors for the Presence of CAD

Variables	OR	95% CI	P-value
Age	1.03	1.00–1.06	0.047
Male	1.34	0.73–2.46	0.348
Smoking	1.76	0.97–3.17	0.061
Dyslipidemia	1.61	0.91–2.84	0.1
Diabetes mellitus	1.18	0.65–2.13	0.584
Lt. PWV	1	1.00–1.00	0.242
EF	0.97	0.93–1.00	0.038
CAC score	1.01	1.01–1.01	< 0.001
BDNF	0.97	0.90–1.05	0.436
Plt	1	0.99–1.01	0.938
ACEi/ARB	0.477	0.25 - 0.91	0.024
CCB	1.24	0.69–2.21	0.474
Statin	1.34	0.71–2.55	0.365
SU	1.39	0.34–5.78	0.649

Significance level was set at P < 0.05. ACEi/ARB: angiotensin-converting-enzyme inhibitor/angiotensin II receptor blocker; BDNF: brain-derived neurotrophic factor; CAC: coronary artery calcification; CAD: coronary artery disease; CCB: calcium channel blocker; CI: confidence interval; EF: ejection fraction; Lt. PWV: left pulse wave velocity; OR: odds ratio; Plt: platelet; SU: sulfonylurea.

Age was independently associated with CAD (odds ratio (OR) 1.03, 95% CI 1.00–1.06, P < 0.05). CAC score showed a strong positive association with CAD (OR 1.01, 95% CI 1.01–1.01, P < 0.01). In contrast, EF showed an inverse correlation with CAD (OR 0.97, 95% CI 0.93–1.00, P < 0.05).

BDNF levels were not independently associated with CAD (OR 0.98, 95% CI 0.89–1.06, P = 0.567). Similarly, MetS, platelet count, inflammatory markers, and other cardiovascular risk factors showed no significant associations. Including CCB, statin, and SU remained not independently associated with CAD. Using ACEi/ARB was also significantly associated with CAD.

### Analysis of confounding factors between BDNF and CAD

The results of the screening for potential confounders are shown in Table 4. To identify potential confounders in the correlation between BDNF and CAD, each candidate variable was added individually to a logistic regression model. With BDNF alone, an inverse correlation was observed between BDNF and CAD (OR 0.94, 95% CI 0.883–0.997, P = 0.039). When platelet count was included in the model, the BDNF estimate changed significantly, with the adjusted OR increasing to 0.976. This represents 62.1% relative change in the exposure coefficient on the log-odds scale. After adjusting for age, the BDNF estimate still changed significantly (42.3% change), while the CAC score showed a moderate change (30.1% change).

**Table 4:** Screening of Potential Confounders for the Association Between Plasma BDNF Levels and CAD

Added covariates	OR (95% CI)	Change-in-estimate (%)	P-value
Model 0 (unadjusted)	0.938 (0.883–0.997)		0.039
Model 0 + Plt	0.976 (0.909–1.05)	62.10%	0.49
Model 0 + age	0.963 (0.902–1.03)	42.30%	0.257
Model 0+ CAC score	0.955 (0.887–1.03)	30.10%	0.224
Model 0 + Lt. PWV	0.948 (0.888–1.01)	17.70%	0.099
Model 0 + HDL-C	0.933 (0.875–0.99)	6.90%	0.03
Model 0 + EF	0.935 (0.877–0.99)	3.10%	0.037
Model 0 + HbA1c	0.937 (0.879–1.00)	0.80%	0.044

Confounding was assessed using the change-in-estimate method. A variable was considered a confounder if its inclusion in the model changed the odds ratio of BDNF by more than 10%. Change-in-estimate (%) was computed using the exposure coefficient from logistic regression (β, i.e., log(odds ratio)) and reflects the percent change in β from the crude model after inclusion of each candidate covariate. Significance level was set at P < 0.05. BDNF: brain-derived neurotrophic factor; CAC: coronary artery calcium; CAD: coronary artery disease; CI: confidence interval; EF: ejection fraction; HbA1c: hemoglobin A1c; HDL-C: high-density lipoprotein cholesterol; Lt. PWV: left pulse wave velocity; OR: odds ratio; Plt: platelet.

Adjustment for left pulse wave velocity (Lt. PWV) yielded a smaller change (17.7% change). HDL-C, EF, and HbA1c produced only minor changes (< 10%) in BDNF estimates, suggesting limited confounding effects.

Next, a cumulative multivariate logistic regression model was constructed based on the confounding factors identified in the single-adjustment analysis (platelet count, age, CAC score, Lt. PWV). The results of the multivariable logistic regression analysis with sequential adjustment are shown in Table 5. In the unadjusted model, higher BDNF levels were inversely correlated with CAD (OR 0.94, 95% CI 0.88–1.00, P = 0.039).

**Table 5:** Multivariable Logistic Regression Analysis of Plasma BDNF Levels for CAD With Sequential Adjustment

Model	Added covariates	OR (95% CI)	P-value
Model 0	Unadjusted	0.938 (0.883–0.997)	0.039
Model 1	Model 0 + Plt	0.976 (0.909–1.044)	0.457
Model 2	Model 1 + age	0.982 (0.915–1.054)	0.617
Model 3	Model 2 + CAC score	0.982 (0.908–1.062)	0.653
Model 4	Model 3 + Lt. PWV	0.980 (0.906–1.061)	0.624

Models were constructed using sequential adjustment. Covariates were added to the model in the order listed in the table. Significance level was set at P < 0.05. BDNF: brain-derived neurotrophic factor; CAC: coronary artery calcium; CAD: coronary artery disease; CI: confidence interval; Lt. PWV: left pulse wave velocity; OR: odds ratio; Plt: platelet.

After sequential adjustment for platelet count, the estimated association between BDNF and CAD moved toward the null value (adjusted OR 0.97), and the P-value also lost significance. Further adjustment for age weakened the association further (OR 0.98). Adjusting for CAC score and Lt. PWV resulted in minimal additional change, with the fully adjusted model yielding an OR of 0.98 (95% CI 0.91–1.06).

## Discussion

The baseline characteristics of the participants showed that age, sex, smoking history, DM, and DLP differed significantly between patients with and without CAD. Laboratory findings demonstrated significantly lower platelet counts in the CAD group, while coronary assessment parameters, such as CAC score and the number of diseased vessels, were markedly higher. Circulating BDNF levels were significantly lower in patients with CAD than in those without CAD (P = 0.039). Furthermore, patients with multivessel disease (VD = 2 or 3) had significantly lower BDNF concentrations than those without multivessel involvement (VD = 0 or 1) (P = 0.03). To evaluate the association between BDNF, cardiovascular risk factors, and CAD, we performed multivariable linear regression analysis. Platelet count showed a significant positive association with BDNF (P < 0.001), whereas no associations were observed between BDNF and cardiovascular risk factors or CAD.

In logistic regression analyses assessing the relationship between CAD and clinical factors, age (P < 0.05), CAC score (P < 0.01), and EF (P = 0.04) were significantly associated with CAD. However, BDNF was not significantly associated with CAD. Because we suspected confounding factors influencing the relationship between BDNF and CAD, we sequentially added potential confounders to the logistic regression models. Platelet count, age, CAC score, and Lt. PWV were identified as relevant confounders. After adjustment for platelet count, the estimated association between BDNF and CAD was attenuated toward the null and became non-significant; additional adjustments produced minimal further change and the association remained non-significant. These findings suggest that platelet count is the principal confounding factor underlying the crude association between BDNF and CAD.

This study shows that BDNF was lower in patients with CAD in unadjusted analyses. The difference in circulating BDNF levels between patients with and without CAD was statistically significant but quantitatively modest. The clinical significance of this absolute difference remains limited, and circulating BDNF should not be interpreted as an established stand-alone biomarker for CAD based on the present findings alone. In addition, this association was no longer significant after adjusting for key confounders, particularly platelet count. Our findings suggest that the crude relationship between BDNF and CAD may be largely attributable to platelet-associated confounding. Our results mainly emphasize that platelet count influences the BDNF and CAD association in a clinically referred CCTA cohort with a relatively intermediate pre-test probability of CAD, and should be considered in future studies evaluating BDNF in cardiovascular disease.

Previous studies have suggested that BDNF levels are low in CAD patients [15]. Meta-analyses have yielded similar findings, though they did not perform corrections using platelet counts [16]. In addition, studies involving clinically referred cohorts with an intermediate pre-test probability of CAD have also indicated lower circulating BDNF levels in patients with the disease. Jin et al reported that among patients with suspected angina who underwent CAG, plasma BDNF levels were lower in those with confirmed CAD than in non-CAD controls [23]. In a cohort study of the general population, individuals with high-normal platelet counts were associated with an increased risk of CAD development during subsequent follow-up [24]. In contrast, a Mendelian randomization study demonstrated that genetically determined platelet count was not associated with the risk of cardiovascular disease [25]. The lack of consistent findings regarding the correlation between CAD, platelets, and BDNF is thought to stem from the characteristics of BDNF and platelets. Circulating BDNF is primarily stored within platelets, with concentrations reported to be 100 to 1,000 times higher than in the central nervous system [26–28]. It is released when platelets are activated by stimuli such as thrombin, collagen, or shear stress [29]. It has also been suggested that the released BDNF itself stimulates platelet aggregation through binding of its receptor, the tropomyosin-related kinase B, inducing platelet secretion [30, 31]. Consequently, BDNF concentration fluctuates significantly with even minor changes in platelet count. Furthermore, BDNF is associated with platelet function and consumption. Additionally, antiplatelet drug use affects BDNF and platelets, with activated platelets increasing BDNF release, while short-term antiplatelet drug administration may reduce BDNF release from platelets into serum [32]. In contrast, serum BDNF concentrations in CAD patients were not associated with stable antiplatelet drug use [33]. In our study, BDNF was relatively strongly correlated with platelet count (β = 0.0266, 95% CI 0.021–0.032) in a clinically referred CCTA cohort with a relatively intermediate pre-test probability of CAD. Platelet count was identified as a relevant confounder. After adjustment for platelet count, the association between BDNF and CAD was attenuated toward the null and was no longer statistically significant. These findings suggest that platelet count should be incorporated into logistic regression models evaluating the association between CAD and BDNF to account for confounding. Moreover, the interplay among CAD, platelets, and BDNF is unlikely to be explained by platelet count alone and may also be affected by factors such as antiplatelet medication use.

### Study limitation

This study is a single-center cross-sectional observational study and cannot evaluate causal relationships between BDNF and CAD or between BDNF and platelets. Detailed symptom data were not systematically collected for the present analysis, and therefore we could not perform a structured analysis of symptoms. It is possible that the non-CAD group includes patients with vasospastic angina. Patients at high risk for CAD may undergo CAG or stress testing in general clinical practice. Consequently, this study includes patients with a relatively intermediate pre-test probability of CAD, raising concerns about selection bias. While patients with a history of CAD were excluded from the cohort, other vascular diseases (stroke, peripheral artery disease) were not included as exclusion criteria. In addition, aspirin use was not systematically collected as a pre-specified variable in the present analysis, and the potential influence of antiplatelet agents, including aspirin, could not be completely excluded. Because medication use in this observational cohort may reflect treatment patterns and underlying clinical indication, the association of ACEi/ARB use with CAD cannot be analyzed causally. Although plasma BDNF was measured in duplicate according to the manufacturer’s instructions, assay-related variability inherent to biomarker measurement could not be completely excluded. In this study, lack of platelet function assessment limits mechanistic interpretation.

### Conclusions

Although BDNF levels were significantly lower in CAD patients, this association disappeared after adjusting for platelet count and other confounding factors. The analysis results suggest that platelets are a major confounding factor between BDNF and CAD. This study indicates that when interpreting BDNF as a biomarker for CAD, correction using platelet data may be necessary.

## Data Availability

The datasets used and/or analyzed during the current study available from the corresponding author on reasonable request.
